# The effects of *IL-27* and *IL-35* gene variation and expression levels on the susceptibility and clinical manifestations of pulmonary tuberculosis

**DOI:** 10.3389/fimmu.2024.1267624

**Published:** 2024-04-16

**Authors:** Lei Gao, Yan-Jun Xiong, Ya-Xue Liang, Peng-Fei Huang, Shuang Liu, Yu Xiao, Qian Huang, Hua Wang, Hui-Mei Wu

**Affiliations:** ^1^ Anhui Geriatric Institute, Department of Geriatric Respiratory and Critical Care Medicine, The First Affiliated Hospital of Anhui Medical University, Hefei, Anhui, China; ^2^ Department of Tuberculosis, Anhui Chest Hospital, Hefei, Anhui, China; ^3^ Department of Public Health, Anhui Medical University, Hefei, China

**Keywords:** pulmonary tuberculosis, single-nucleotide polymorphisms, IL-27, IL-35, infectious diseases

## Abstract

Inflammatory cytokines have crucial roles in the pathogenesis of tuberculosis (TB), and interleukin (IL)-27 and IL-35 have a pro-inflammatory and anti-inflammatory effect on many diseases, including infectious diseases. Therefore, we evaluated the relationship between *IL-27* and *IL-35* gene polymorphism, expression levels, and pulmonary TB (PTB) susceptibility. Nine single-nucleotide polymorphisms (SNPs) in the *IL-27* gene (rs181206, rs153109, and rs17855750) and the *IL-35* gene (rs4740, rs428253, rs9807813, rs2243123, rs2243135, and rs568408) were genotyped by the SNPscan technique in 497 patients with PTB and 501 controls. There was no significant difference regarding the genotype and allele frequencies of the above SNPs in the *IL-27* and *IL-35* genes between patients with PTB and controls. Haplotype analysis showed that the frequency of the GAC haplotype in the *IL-35* gene was significantly decreased in patients with PTB when compared to controls (*p =* 0.036). Stratified analysis suggested that the frequency of the *IL-27* rs17855750 GG genotype was significantly increased in patients with PTB with fever. Moreover, the lower frequency of the *IL-35* rs568408 GA genotype was associated with drug-induced liver injury in patients with PTB. The *IL-35* rs428253 GC genotype, as well as the rs4740 AA genotype and A allele, showed significant relationships with hypoproteinemia in patients with PTB. When compared with controls, the IL-27 level was significantly increased in patients with PTB. Taken together, *IL-35* gene variation might contribute to a protective role on the susceptibility to PTB, and *IL-27* and *IL-35* gene polymorphisms were associated with several clinical manifestations of patients with PTB.

## Introduction

As a serious infectious disease, tuberculosis (TB) is mainly caused by *Mycobacterium tuberculosis* (MTB) infection and is a leading cause of death from a single infectious disease alongside COVID-19. The latest report from the World Health Organization (WHO) showed that there were approximately 10.6 million new cases of TB globally and approximately 0.78 million new TB cases in China in 2021 (1). After infection with MTB, this pathogen is usually not eliminated immediately and might exist in macrophages over a long period due to its ability to evade the host immune system (2). Therefore, the development of TB was affected by the interaction of multiple factors. A previous study found that about a quarter of the global population was infected with MTB, but only 10% of infected people would eventually develop active TB (3). Combined with other studies, it has been found that certain host factors, including genetic factors, malnutrition, and HIV infection, could significantly influence the susceptibility to MTB infection through regulating the host immune status (4, 5).

In recent years, genome-wide association studies and transcriptomic association studies have identified a number of variant loci that affected pulmonary TB (PTB) susceptibility, while the genetic factors that affected the susceptibility to PTB were not fully understood (6). Notably, genetic variations in several immune-related genes were shown to affect the degree of host resistance to MTB infection, disease severity, and drug resistance (7, 8). Previous studies found that polymorphisms in *interleukin (IL)-13*, *IL-13RA1*, *IL-13RA2*, and *IL-23R* genes were involved in the genetic background of PTB and some clinical features in a Chinese Han population (9, 10). Therefore, it was of great significance to further clarify the effects of immune-related gene genetic variations in PTB.

Studies showed that the differentiation of naive T cells into Thl lymphocytes, which was very important for a successful anti-TB immune response, required the presence of the IL-12 family cytokines (IL-27, IL-35, and IL-12) (11). During MTB infection, IL-27 could promote Th1 cell differentiation by STAT1 phosphorylation against MTB (12). In addition, IL-27 signaling was indispensable for the activation of Tr1 cells and induction of IL-10 expression through STAT3 phosphorylation. It also inhibited immune response to MTB infection and increased disease severity (12). IL-35 consisted of the IL-12 subunit α chain (IL-12A/P35) and EBI3, and expressed in many immune cells (13). Kong et al. demonstrated that serum IL-35 level was increased in patients with active PTB (14), and another study found that the mRNA expression levels of two subunits (p35 and Ebi3) of IL-35 by circulating B cells were also increased in active PTB (15). Therefore, these inflammatory cytokines played essential roles in the pathogenesis of PTB.

The significance of genetic variations in *IL-27* and *IL-35* genes has been reported in many diseases (16, 17). However, there were few studies on *IL-27* gene variation in PTB, and no study on the gene variation of *IL-35* in PTB was performed. Candidate genetic association studies remained a useful way to assess the association of gene variation with host immune response to MTB stimulation. Hence, we performed this case–control study to evaluate the possible association between *IL-27* and *IL-35* gene polymorphism, expression levels, and PTB susceptibility in a Chinese Han population.

## Materials and methods

### Patients with PTB and controls

In this study, we enrolled patients with PTB from the Anhui Chest Hospital and collected normal controls from a health examination center. Subjects consisted of unrelated Chinese Han individuals. Patients with multiple clinical manifestations, such as the detection of acid-fast bacilli in sputum smear samples and the results of MTB cultures and chest radiograph, were confirmed to have PTB by an experienced senior physician according to the Diagnosis for PTB (WS 288-2017) and the Classification of TB (WS 196-2017) from the Health Industry Standard of the People’s Republic of China. Patients with PTB who are HIV-positive and who have hepatitis virus infection, cancer, autoimmune diseases, and other infectious diseases were excluded from this study. At the same time, the control group was required to be healthy volunteers with normal chest imaging examination and with no history of TB, infectious diseases, lung disease, and bacterial or viral infection.

Our study was approved by the Ethics Committee of the Anhui Chest Hospital, and the written informed consent of every subject was obtained. Then, peripheral blood (3–5 mL) of subjects was collected for extracting DNA with the Flexi Gene-DNA Kit (Qiagen, Valencia, CA). Meanwhile, we obtained the clinical characteristics, such as drug resistance, fever, pulmonary infection, drug-induced liver injury (DILI), and sputum smear, from the hospital medical record system.

### SNP selection and genotyping

We searched Ensembl genome browser 85 and the CHBS_1000g database to obtain genetic variations of the *IL-27* and *IL-35* genes and then chose the TagSNPs using the Haploview 4.0 software (Cambridge, MA, USA). The candidate TagSNPs were selected based on three criteria: (1) minor allele frequency (MAF) > 0.05 in a Chinese Han Beijing population; (2) an *r* ^2^ cutoff of 0.8 for linkage disequilibrium; and (3) the flanking 2.0-kb regions of *IL-27* and *IL-35* genes. Ultimately, nine tagSNPs were selected in this study, namely, three tagSNPs in the *IL-27* gene (rs181206, rs153109, and rs17855750) and six tagSNPs in the *IL-35* gene (for *IL-12A*: rs2243123, rs2243135, and rs568408; for *EBI3*: rs428253, rs4740, and rs9807813).

Subsequently, this study genotyped all single-nucleotide polymorphisms (SNPs) in *IL-27* and *IL-35* genes by the SNPscan technique, with technical support from the Center for Genetic and Genomic Analysis, Genesky Biotechnologies Inc. (Shanghai).

### Enzyme-linked immunosorbent assay

Plasma was extracted from peripheral blood (at 3,000 rpm for 10 min at room temperature) and stored at −80°C until processed. The expression levels of IL-27 and IL-35 were determined using an enzyme-linked immunosorbent assay (ELISA) kit (ELK1572 and 4360164, and ELK2745 and 4362411, ELK Biotechnology) according to standard experimental procedures. The IL-27 and IL-35 levels were expressed as nanograms per milliliter.

### Statistical analysis

The genotype frequencies of all SNPs among the control group were evaluated for Hardy–Weinberg equilibrium (HWE) by chi-square (*χ* ^2^). Genotype and allele frequencies between patients with PTB and controls were assessed with logistic regression.

The relationship between *IL-27* and *IL-35* gene polymorphisms and PTB susceptibility was shown as odds ratio (OR) and 95% confidence interval (95% CI). The association between each SNP with clinical manifestations was evaluated using *χ* ^2^. Two genetic models (dominant model and negative model) were applied to analyze the association between SNPs and PTB risk, and the SHEsis software was used for haplotype analysis. The IL-27 and IL-35 expression levels were shown as medians [interquartile range (IQR)], and the comparisons of IL-27 and IL-35 expression levels were analyzed by Mann–Whitney *U* test and Kruskal–Wallis *H* test. Statistical analysis was performed using SPSS 23.0, and *p*-value less than 0.05 was regarded as statistically significant.

## Results

### Demographic data and clinical manifestations of study subjects

A total of 998 study subjects were included in this study, including 497 patients with PTB and 501 controls. Among patients with PTB, the average age of 313 male and 184 female patients was 44.72 ± 18.03 years. Among controls, the average age of 300 male and 201 female patients was 44.42 ± 3.51 years. Distribution differences regarding sex and age between these two groups were not statistically significant. The distribution of several major clinical manifestations in patients with PTB is shown in **Table 1**.

**Table 1:** The main demographic and clinical characteristics of patients with PTB and controls.

Characteristics	Patients with PTB (*n* = 497)	Controls (*n* = 501)
Demographic characteristics		
Age (years)	44.72 ± 18.03	44.42 ± 3.51
Sex (male/female)	313/184	300/201
Clinical characteristics^a^		
Fever (+/−)	83/413	NA
Drug resistance (+/−)	74/422	NA
DILI (+/−)	68/428	NA
Pulmonary infection (+/−)	61/435	NA
Hypoproteinemia (+/−)	41/455	NA
Sputum smear-positive (+/−)	140/313	NA

NA, not applicable; +/−, with/without; ^a^part of the study subjects’ data are missing.

### Association of SNPs in *IL-27* and *IL-35* genes with PTB susceptibility

The allele and genotype frequencies of nine SNPs in *IL-27* and *IL-35* genes are presented in **Table 2**. Apart from the rs181206 variant, the genotype frequencies of other SNPs in the control group were consistent with genetic equilibrium via the HWE test (*p* > 0.05). Then, the rs181206 variant was not included for analysis.

**Table 2:** *IL-27* and *IL-35* genes’ genotype and allele frequencies among patients with PTB and controls.

SNP	Analyze model		Patients with PTB	Controls	*p*-value	OR (95% CI)
IL-27						
rs153109	Genotypes	CC	68 (13.68)	63 (12.57)	0.275	1.246 (0.840,1.848)
		TC	241 (48.49)	221 (44.11)	0.092	1.259 (0.963,1.644)
		TT	188 (37.83)	217 (43.31)	Reference	
	Alleles	C	377 (37.93)	347 (34.63)	0.126	1.153 (0.961,1.384)
		T	617 (62.07)	655 (65.37)	Reference	
	Dominant model	TT	188 (37.83)	217 (43.31)	0.078	0.796 (0.618,1.26)
		TC+CC	309 (62.17)	284 (56.69)	Reference	
	Recessive model	CC	68 (13.68)	63 (12.57)	0.605	1.102 (0.763,1.592)
		TC+TT	429 (86.32)	438 (87.43)	Reference	
rs17855750	Genotypes	CC	5 (1.01)	3 (0.6)	0.465	1.708 (0.406,7.196)
		AC	93 (18.71)	89 (17.76)	0.675	1.071 (0.776,1.478)
		AA	399 (80.28)	409 (81.64)	Reference	
	Alleles	C	103 (10.36)	95 (9.48)	0.510	1.104 (0.823,1.481)
		A	891 (89.64)	907 (90.52)	Reference	
	Dominant model	AA	399 (80.28)	409 (81.64)	0.586	0.916 (0.668,1.256)
		AC+CC	98 (19.72)	92 (18.36)	Reference	
	Recessive model	CC	5 (1.01)	3 (0.6)	0.471	1.687 (0.401,7.097)
		AC+AA	492 (98.99)	498 (99.4)	Reference	
IL-35						
rs2243123	Genotypes	CC	3 (0.6)	2 (0.4)	0.654	1.507 (0.251,9.066)
		TC	77 (15.49)	80 (15.97)	0.848	0.967 (0.688,1.360)
		TT	417 (83.9)	419 (83.63)	Reference	
	Alleles	C	83 (8.35)	84 (8.38)	0.979	0.996 (0.725,1.367)
		T	911 (91.65)	918 (91.62)	Reference	
	Dominant model	TT	417 (83.9)	419 (83.63)	0.908	0.983 (0.742,1.060)
		TC+CC	80 (16.1)	82 (16.37)	Reference	
	Recessive model	CC	3 (0.6)	2 (0.4)	0.647	1.515 (0.252,9.107)
		TC+TT	494 (99.4)	499 (99.6)	Reference	
rs2243135	Genotypes	CC	11 (2.21)	10 (2)	0.741	1.158 (0.486,2.761)
		GC	144 (28.97)	131 (26.15)	0.306	1.157 (0.875,1.530)
		GG	342 (68.81)	360 (71.86)	Reference	
	Alleles	C	166 (16.7)	151 (15.07)	0.319	1.130 (0.889,1.437)
		G	828 (83.3)	851 (84.93)	Reference	
	Dominant model	GG	342 (68.81)	360 (71.86)	0.293	0.864 (0.658,1.134)
		GC+CC	155 (31.19)	141 (28.14)	Reference	
	Recessive model	CC	11 (2.21)	10 (2)	0.811	1.111 (0.468,2.641)
		GC+GG	486 (97.79)	491 (98)	Reference	
rs568408	Genotypes	AA	9 (1.81)	11 (2.2)	0.613	0.794 (0.325,1.939)
		GA	110 (22.13)	123 (24.55)	0.347	0.868 (0.647,1.166)
		GG	378 (76.06)	367 (73.25)	Reference	
	Alleles	A	128 (12.88)	145 (14.47)	0.300	0.874 (0.676,1.128)
		G	866 (87.12)	857 (85.53)	Reference	
	Dominant model	GG	495 (99.6)	502 (100.2)	0.458	1.110 (0.842,1.464)
		GA+AA	119 (23.94)	134 (26.75)	Reference	
	Recessive model	AA	9 (1.81)	11 (2.2)	0.665	0.822 (0.337,2.000)
		GA+GG	488 (98.19)	490 (97.8)	Reference	
rs428253	Genotypes	CC	25 (5.03)	23 (4.59)	0.668	1.137 (0.632,2.046)
		GC	168 (33.8)	160 (31.94)	0.492	1.098 (0.841,1.435)
		GG	304 (61.17)	318 (63.47)	Reference	
	Alleles	C	218 (21.93)	206 (20.56)	0.453	1.086 (0.876,1.345)
		G	776 (78.07)	796 (79.44)	Reference	
	Dominant model	GG	304 (61.17)	318 (63.47)	0.452	0.906 (0.702,1.171)
		GC+CC	193 (38.83)	183 (36.53)	Reference	
	Recessive model	CC	25 (5.03)	23 (4.59)	0.746	1.101 (0.616,1.967)
		GC+GG	472 (94.97)	478 (95.41)	Reference	
rs4740	Genotypes	AA	94 (18.91)	115 (22.95)	0.169	0.781 (0.550,1.111)
		GA	244 (49.09)	234 (46.71)	0.983	0.997 (0.749,1.326)
		GG	159 (31.99)	152 (30.34)	Reference	
	Alleles	A	432 (43.46)	464 (46.31)	0.201	0.891 (0.747,1.063)
		G	562 (56.54)	538 (53.69)	Reference	
	Dominant model	GG	159 (31.99)	152 (30.34)	0.573	1.080 (0.826,1.412)
		GA+AA	338 (68.01)	349 (69.66)	Reference	
	Recessive model	AA	94 (18.91)	115 (22.95)	0.117	0.783 (0.576,1.063)
		GA+GG	403 (81.09)	386 (77.05)	Reference	
rs9807813	Genotypes	TT	17 (3.42)	22 (4.39)	0.437	0.773 (0.403,1.481)
		CT	146 (29.38)	145 (28.94)	0.961	1.007 (0.765,1.326)
		CC	334 (67.2)	334 (66.67)	Reference	
	Alleles	T	180 (18.11)	189 (18.86)	0.665	0.951 (0.759,1.193)
		C	814 (81.89)	813 (81.14)	Reference	
	Dominant model	CC	334 (67.2)	334 (66.67)	0.857	1.025 (0.787,1.334)
		CT+TT	163 (32.8)	167 (33.33)	Reference	
	Recessive model	TT	17 (3.42)	22 (4.39)	0.429	0.771 (0.404,1.470)
		CT+CC	480 (96.58)	479 (95.61)	Reference	

Our results found no statistically significant difference in allele and genotype frequencies of *IL-27* gene rs153109 and rs17855750 polymorphisms among patients with PTB and controls. Similarly, the distribution difference in alleles and genotypes of rs2243123, rs2243135, rs568408, rs428253, rs4740, and rs9807813 variants in the *IL-35* gene was not statistically significant (**Table 2**). Furthermore, two genetic models (dominant model and recessive model) were applied to assess the relationship between the above SNPs with PTB susceptibility, while the results showed no significant association (**Table 2**).

### Haplotype analysis

IL-35 consisted of an IL-12 subunit α chain (IL-12A/P35) and EBI3, and the polymorphisms in the *IL-35* gene were respectively from *IL-12A* (rs2243123, rs2243135, and rs568408) and *EBI3* (rs428253, rs4740, and rs9807813). Hence, we respectively used the SHEsis software to construct the haplotype of *IL-27* gene rs153109–rs17855750, *IL-35* gene rs2243123–rs2243135–rs568408, and *IL-35* gene rs428253–rs4740–rs9807813, and the main haplotypes with a frequency of more than 3% are shown in **Table 3**. We suggested that the GAC haplotype (representing rs428253–rs4740–rs9807813) of the *IL-35* gene was a protective factor against PTB susceptibility (*p =* 0.036, OR *=* 0.751). However, other haplotypes showed no statistical association with PTB susceptibility.

**Table 3:** Haplotype analysis of *IL-27* and *IL-35* genes in patients with PTB.

Haplotype	Patients with PTB	Controls	*p*-value	OR (95% CI)
*IL-27* rs153109–rs17855750				
CA	275.38 (0.277)	256.08 (0.256)	0.294	1.112 (0.912,1.357)
CC	101.62 (0.102)	90.92 (0.091)	0.395	1.138 (0.845,1.532)
TA	615.62 (0.619)	650.92 (0.650)	0.137	0.871 (0.725,1.045)
*IL-35* rs2243123–rs2243135–rs568408				
CCG	77.51 (0.078)	74.35 (0.074)	0.767	1.051 (0.755,1.464)
TCG	87.77 (0.088)	76.59 (0.076)	0.347	1.166 (0.847,1.606)
TGA	121.79 (0.123)	135.33 (0.135)	0.387	0.891 (0.685,1.158)
TGG	700.72 (0.705)	706.06 (0.705)	0.916	0.990 (0.815,1.202)
*IL-35* rs428253–rs4740–rs9807813				
CAC	144.45 (0.145)	135.24 (0.135)	0.487	1.094 (0.849,1.409)
CGC	68.14 (0.069)	68.40 (0.068)	0.964	1.008 (0.712,1.427)
GAC	107.55 (0.108)	139.76 (0.139)	**0.036**	0.751 (0.574,0.982)
GAT	174.59 (0.176)	186.63 (0.186)	0.559	0.934 (0.744,1.174)
GGC	493.86 (0.497)	469.60 (0.469)	0.184	1.127 (0.945,1.343)

Frequency < 0.03 in both controls and patients with PTB has been dropped.

Bold value means p < 0.05.

### Association of *IL-27* and *IL-35* gene polymorphisms and clinical manifestations among patients with PTB

This study also examined whether polymorphisms in *IL-27* and *IL-35* genes affected the clinical manifestations of PTB, and the results are summarized in **Table 4**. In the *IL-27* gene, the increased frequency of the rs17855750 GG genotype was found in patients with PTB with fever when compared to the patients without fever (*p =* 0.001). For the *IL-35* gene, the rs568408 GA genotype frequency in patients with PTB with DILI was significantly lower than that in the patients without DILI (*p =* 0.006). In addition, the decreased frequencies of the rs428253 GC genotype, rs4740 AA genotype, and A allele were significantly related to hypoproteinemia in patients with PTB (*p =* 0.026, *p =* 0.008, and *p =* 0.007).

**Table 4:** *IL-27* and *IL-35* gene polymorphisms and the clinical manifestations in patients with PTB.

SNP	Allele	Clinical features	Group	Genotype			*p-*value	Allele		*p*-value
	(Mm)			MM	Mm	mm		M	m	
IL-27										
rs153109	T/C	Fever	+	25 (30.12)	44 (53.01)	14 (16.87)	0.252	94 (56.63)	72 (43.37)	0.111
			−	163 (39.47)	196 (47.46)	54 (13.08)		522 (63.2)	304 (36.8)	
		Drug resistance	+	25 (33.78)	36 (48.65)	13 (17.57)	0.513	86 (58.11)	62 (41.89)	0.278
			−	163 (38.63)	204 (48.34)	55 (13.03)		530 (62.8)	314 (37.2)	
		DILI	+	20 (29.41)	36 (52.94)	12 (17.65)	0.256	76 (55.88)	60 (44.12)	0.108
			−	168 (39.25)	204 (47.66)	56 (13.08)		540 (63.08)	316 (36.92)	
		Pulmonary infection	+	19 (31.15)	29 (47.54)	13 (21.31)	0.151	67 (54.92)	55 (45.08)	0.081
			−	169 (38.85)	211 (48.51)	55 (12.64)		549 (63.1)	321 (36.9)	
		Hypoproteinemia	+	10 (24.39)	26 (63.41)	5 (12.2)	0.116	46 (56.1)	36 (43.9)	0.242
			−	178 (39.12)	214 (47.03)	63 (13.85)		570 (62.64)	340 (37.36)	
		Sputum smear-positive	+	45 (32.14)	75 (53.57)	20 (14.29)	0.257	165 (58.93)	115 (41.07)	0.251
			−	125 (39.94)	144 (46.01)	44 (14.06)		394 (62.94)	232 (37.06)	
rs17855750	A/C	Fever	+	65 (78.31)	14 (16.87)	4 (4.82)	**0.001**	144 (86.75)	22 (13.25)	0.184
			−	333 (80.63)	79 (19.13)	1 (0.24)		745 (90.19)	81 (9.81)	
		Drug resistance	+	58 (78.38)	16 (21.62)	0 (0)	0.523	132 (89.19)	16 (10.81)	0.853
			−	340 (80.57)	77 (18.25)	5 (1.18)		757 (89.69)	87 (10.31)	
		DILI	+	56 (82.35)	12 (17.65)	0 (0)	0.641	124 (91.18)	12 (8.82)	0.521
			−	342 (79.91)	81 (18.93)	5 (1.17)		765 (89.37)	91 (10.63)	
		Pulmonary infection	+	50 (81.97)	10 (16.39)	1 (1.64)	0.776	110 (90.16)	12 (9.84)	0.832
			−	348 (80)	83 (19.08)	4 (0.92)		779 (89.54)	91 (10.46)	
		Hypoproteinemia	+	36 (87.8)	5 (12.2)	0 (0)	0.408	77 (93.9)	5 (6.1)	0.184
			−	362 (79.56)	88 (19.34)	5 (1.1)		812 (89.23)	98 (10.77)	
		Sputum smear-positive	+	113 (80.71)	26 (18.57)	1 (0.71)	0.955	252 (90)	28 (10)	0.804
			−	250 (79.87)	60 (19.17)	3 (0.96)		560 (89.46)	66 (10.54)	
IL-35										
rs2243123	T/C	Fever	+	72 (86.75)	11 (13.25)	0 (0)	0.597	155 (93.37)	11 (6.63)	0.375
			−	344 (83.29)	66 (15.98)	3 (0.73)		754 (91.28)	72 (8.72)	
		Drug resistance	+	64 (86.49)	9 (12.16)	1 (1.35)	0.471	137 (92.57)	11 (7.43)	0.656
			−	352 (83.41)	68 (16.11)	2 (0.47)		772 (91.47)	72 (8.53)	
		DILI	+	57 (83.82)	11 (16.18)	0 (0)	0.779	125 (91.91)	11 (8.09)	0.899
			−	359 (83.88)	66 (15.42)	3 (0.7)		784 (91.59)	72 (8.41)	
		Pulmonary infection	+	52 (85.25)	9 (14.75)	0 (0)	0.793	113 (92.62)	9 (7.38)	0.673
			−	364 (83.68)	68 (15.63)	3 (0.69)		796 (91.49)	74 (8.51)	
		Hypoproteinemia	+	35 (85.37)	6 (14.63)	0 (0)	0.859	76 (92.68)	6 (7.32)	0.720
			−	381 (83.74)	71 (15.6)	3 (0.66)		833 (91.54)	77 (8.46)	
		Sputum smear-positive	+	118 (84.29)	21 (15)	1 (0.71)	0.992	257 (91.79)	23 (8.21)	0.963
			−	263 (84.03)	48 (15.34)	2 (0.64)		574 (91.69)	52 (8.31)	
rs2243135	G/C	Fever	+	53 (63.86)	29 (34.94)	1 (1.2)	0.345	135 (81.33)	31 (18.67)	0.439
			−	289 (69.98)	114 (27.6)	10 (2.42)		692 (83.78)	134 (16.22)	
		Drug resistance	+	56 (75.68)	17 (22.97)	1 (1.35)	0.387	129 (87.16)	19 (12.84)	0.179
			−	286 (67.77)	126 (29.86)	10 (2.37)		698 (82.7)	146 (17.3)	
		DILI	+	44 (64.71)	22 (32.35)	2 (2.94)	0.694	110 (80.88)	26 (19.12)	0.402
			−	298 (69.63)	121 (28.27)	9 (2.1)		717 (83.76)	139 (16.24)	
		Pulmonary infection	+	46 (75.41)	14 (22.95)	1 (1.64)	0.507	106 (86.89)	16 (13.11)	0.265
			−	296 (68.05)	129 (29.66)	10 (2.3)		721 (82.87)	149 (17.13)	
		Hypoproteinemia	+	28 (68.29)	11 (26.83)	2 (4.88)	0.474	67 (81.71)	15 (18.29)	0.673
			−	314 (69.01)	132 (29.01)	9 (1.98)		760 (83.52)	150 (16.48)	
		Sputum smear-positive	+	98 (70.00)	37 (26.43)	5 (3.57)	0.230	233 (83.21)	47 (16.79)	0.806
			−	216 (69.01)	93 (29.71)	4 (1.28)		525 (83.87)	101 (16.13)	
rs568408	G/A	Fever	+	64 (77.11)	17 (20.48)	2 (2.41)	0.844	145 (87.35)	21 (12.65)	0.915
			−	313 (75.79)	93 (22.52)	7 (1.69)		719 (87.05)	107 (12.95)	
		Drug resistance	+	52 (70.27)	21 (28.38)	1 (1.35)	0.370	125 (84.46)	23 (15.54)	0.299
			−	325 (77.01)	89 (21.09)	8 (1.90)		739 (87.56)	105 (12.44)	
		DILI	+	55 (80.88)	9 (13.24)	4 (5.88)	**0.006**	119 (87.50)	17 (12.50)	0.880
			−	322 (75.23)	101 (23.6)	5 (1.17)		745 (87.03)	111 (12.97)	
		Pulmonary infection	+	48 (78.69)	13 (21.31)	0 (0)	0.509	109 (89.34)	13 (10.66)	0.429
			−	329 (75.63)	97 (22.3)	9 (2.07)		755 (86.78)	115 (13.22)	
		Hypoproteinemia	+	35 (85.37)	5 (12.2)	1 (2.44)	0.270	75 (91.46)	7 (8.54)	0.218
			−	342 (75.16)	105 (23.08)	8 (1.76)		789 (86.70)	121 (13.30)	
		Sputum smear-positive	+	115 (82.14)	24 (17.14)	1 (0.71)	0.151	254 (90.71)	26 (9.29)	0.052
			−	232 (74.12)	75 (23.96)	6 (1.92)		539 (86.10)	87 (13.90)	
rs428253	G/C	Fever	+	54 (65.06)	27 (32.53)	2 (2.41)	0.441	135 (81.33)	31 (18.67)	0.274
			−	250 (60.53)	140 (33.9)	23 (5.57)		640 (77.48)	186 (22.52)	
		Drug resistance	+	46 (62.16)	24 (32.43)	4 (5.41)	0.964	116 (78.38)	32 (21.62)	0.936
			−	258 (61.14)	143 (33.89)	21 (4.98)		659 (78.08)	185 (21.92)	
		DILI	+	43 (63.24)	20 (29.41)	5 (7.35)	0.520	106 (77.94)	30 (22.06)	0.955
			−	261 (60.98)	147 (34.35)	20 (4.67)		669 (78.15)	187 (21.85)	
		Pulmonary infection	+	33 (54.10)	24 (39.34)	4 (6.56)	0.456	90 (73.77)	32 (26.23)	0.214
			−	271 (62.30)	143 (32.87)	21 (4.83)		685 (78.74)	185 (21.26)	
		Hypoproteinemia	+	32 (78.05)	6 (14.63)	3 (7.32)	**0.026**	70 (85.37)	12 (14.63)	0.098
			−	272 (59.78)	161 (35.38)	22 (4.84)		705 (77.47)	205 (22.53)	
		Sputum smear-positive	+	85 (60.71)	51 (36.43)	4 (2.86)	0.522	221 (78.93)	59 (21.07)	0.743
			−	191 (61.02)	106 (33.87)	16 (5.11)		488 (77.96)	138 (22.04)	
rs4740	G/A	Fever	+	31 (37.35)	36 (43.37)	16 (19.28)	0.468	98 (59.04)	68 (40.96)	0.479
			−	128 (30.99)	207 (50.12)	78 (18.89)		463 (56.05)	363 (43.95)	
		Drug resistance	+	20 (27.03)	40 (54.05)	14 (18.92)	0.565	80 (54.05)	68 (45.95)	0.506
			−	139 (32.94)	203 (48.1)	80 (18.96)		481 (56.99)	363 (43.01)	
		DILI	+	25 (36.76)	25 (36.76)	18 (26.47)	0.071	75 (55.15)	61 (44.85)	0.722
			−	134 (31.31)	218 (50.93)	76 (17.76)		486 (56.78)	370 (43.22)	
		Pulmonary infection	+	26 (42.62)	24 (39.34)	11 (18.03)	0.151	76 (62.30)	46 (37.70)	0.172
			−	133 (30.57)	219 (50.34)	83 (19.08)		485 (55.75)	385 (44.25)	
		Hypoproteinemia	+	22 (53.66)	14 (34.15)	5 (12.2)	**0.008**	58 (70.73)	24 (29.27)	**0.007**
			−	137 (30.11)	229 (50.33)	89 (19.56)		503 (55.27)	407 (44.73)	
		Sputum smear-positive	+	44 (31.43)	69 (49.29)	27 (19.29)	0.939	157 (56.07)	123 (43.93)	0.788
			−	100 (31.95)	157 (50.16)	56 (17.89)		357 (57.03)	269 (42.97)	
rs9807813	C/T	Fever	+	60 (72.29)	19 (22.89)	4 (4.82)	0.300	139 (83.73)	27 (16.27)	0.491
			−	273 (66.10)	127 (30.75)	13 (3.15)		673 (81.48)	153 (18.52)	
		Drug resistance	+	45 (60.81)	28 (37.84)	1 (1.35)	0.157	118 (79.73)	30 (20.27)	0.467
			−	288 (68.25)	118 (27.96)	16 (3.79)		694 (82.23)	150 (17.77)	
		DILI	+	47 (69.12)	19 (27.94)	2 (2.94)	0.923	113 (83.09)	23 (16.91)	0.688
			−	286 (66.82)	127 (29.67)	15 (3.50)		699 (81.66)	157 (18.34)	
		Pulmonary infection	+	45 (73.77)	13 (21.31)	3 (4.92)	0.291	103 (84.43)	19 (15.57)	0.431
			−	288 (66.21)	133 (30.57)	14 (3.22)		709 (81.49)	161 (18.51)	
		Hypoproteinemia	+	28 (68.29)	12 (29.27)	1 (2.44)	0.934	68 (82.93)	14 (17.07)	0.793
			−	305 (67.03)	134 (29.45)	16 (3.52)		744 (81.76)	166 (18.24)	
		Sputum smear-positive	+	88 (62.86)	47 (33.57)	5 (3.57)	0.398	223 (79.64)	57 (20.36)	0.193
			−	217 (69.33)	87 (27.80)	9 (2.88)		521 (83.23)	105 (16.77)	

Bold value means p < 0.05; +, with; −, without.

### Association of IL-27 and IL-35 expression levels with patients with PTB

**Figure 1:**
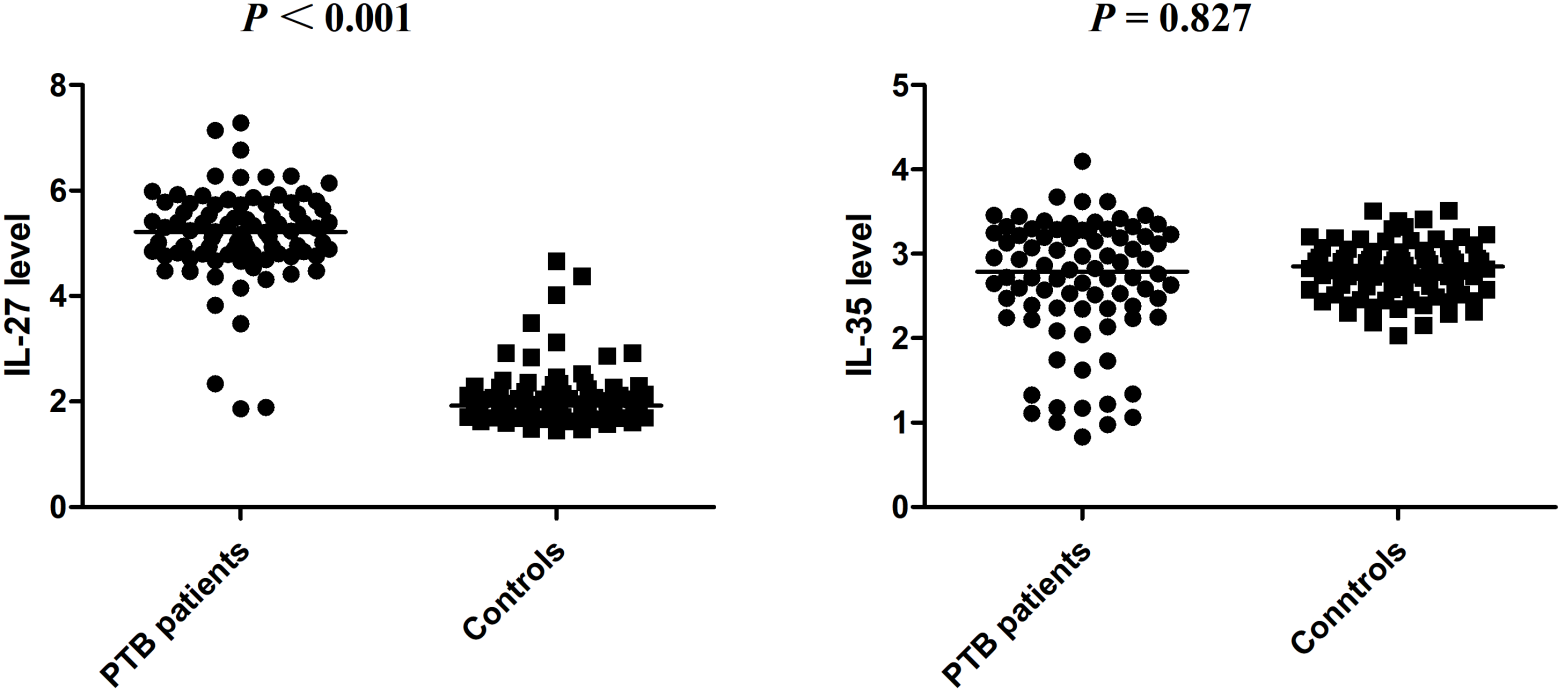
The expression levels of IL-27 and IL-35 in patients with PTB and normal controls.

We then examined the IL-27 and IL-35 plasma levels in 84 patients with PTB (namely, 59 male and 25 female patients; average age: 50.73 ± 19.24 years) and 84 controls (namely, 57 male and 27 female patients; average age: 49.35 ± 6.12 years). The IL-27 plasma level of patients with PTB and controls were 5.217 (4.791, 5.731) ng/mL and 1.923 (1.748, 2.233) ng/mL, respectively. The IL-35 plasma level of patients with PTB and controls were 2.788 (2.349, 3.244) ng/mL and 2.852 (2.579, 3.017) ng/mL, respectively. We found that when compared to the control group, the plasma level of IL-27 was abnormally elevated in patients with PTB (*p* < 0.001), while the plasma level of IL-35 was not significantly different (*p =* 0.827) (**Figure 1**).

The possible associations between IL-27 and IL-35 expression levels and several clinical features were also assessed among patients with PTB. The results found that the IL-27 and IL-35 expression levels were not associated with fever, drug resistance, DILI pulmonary infection, hypoproteinemia, and sputum smear-positive in patients with PTB (**Table 5**).

**Table 5:** The IL-27 and IL-35 levels and the main clinical manifestations among patients with PTB.

Clinical features	Group	IL-27 level	*p-*value	IL-35 level	*p-*value
Fever	+	5.384 (4.830,5.606)	0.497	2.827 (2.278,3.257)	0.779
	−	5.150 (4.759,5.749)		2.788 (2.352,3.253)	
Drug resistance	+	5.394 (5.131,5.732)	0.525	2.721 (0.830,3.217)	0.589
	−	5.209 (4.765,5.746)		2.811 (2.353,3.266)	
DILI	+	5.544 (5.134,5.765)	0.132	3.199 (2.822,3.290)	0.111
	−	5.131 (4.759,5.730)		2.721 (2.299,3.224)	
Pulmonary infection	+	5.385 (5.103,5.772)	0.213	2.978 (2.708,3.421)	0.056
	−	5.131 (4.752,5.131)		2.721 (2.246,3.217)	
Hypoproteinemia	+	5.13 (4.765,5.585)	0.996	2.811 (2.251,3.191)	0.978
	−	5.243 (4.797,5.746)		2.766 (2.361,3.266)	
Sputum smear-positive	+	5.042 (4.791,5.042)	0.265	2.948 (2.606,3.289)	0.094
	−	5.372 (4.758,5.762)		2.678 (2.123,3.235)	

+: with; -: without.

### Association of IL-27 and IL-35 expression levels with their genotype frequencies in patients with PTB

We also investigated the relationship between IL-27 and IL-35 expression levels and their respective genotypes in 50 patients with PTB. However, no statistically significant association was found in this study (**Table 6**).

**Table 6:** Association between IL-27 and IL-35 levels and their genotype frequencies among patients with PTB.

IL-27 SNP	N	IL-27 level	p-value
rs153109			0.164
CC	10	5.163 (4.475,5.568)	
TC	25	5.302 (4.793,5.794)	
TT	15	4.848 (4.1478,5.372)	
rs17855750			0.413
CC	2	5.520 (5.400,5.641)	
AC	11	5.337 (4.692,5.869)	
AA	37	4.927 (4.623,5.672)	
*IL-35* SNP	N	IL-35 level	*p-*value
rs2243123			0.930
TC	9	2.865 (2.657,3.117)	
TT	41	3.058 (2.372,3.338)	
rs2243135			0.560
GC	16	2.766 (2.533,3.271)	
CC	34	3.064 (2.453,3.331)	
rs568408			0.985
AA	1	2.935	
GA	10	2.959 (2.453,3.380)	
GG	39	2.902 (2.516,3.298)	
rs428253			0.988
CC	1	2.865	
GC	20	2.996 (2.363,3.360)	
GG	29	2.902 (2.562,3.285)	
rs4740			0.188
AA	11	3.117 (2.865,3.352)	
GA	26	2.688 (2.382,3.254)	
GG	13	3.045 (2.524,3.439)	
rs9807813			0.897
CT	19	3.058 (2.655,3.273)	
CC	31	2.714 (2.474,3.362)	

## Discussion

The host immune response was an important factor affecting the development of PTB. The innate and adaptive immune systems of the body were consecutively activated after MTB infection, and immune cells released a large number of cytokines to exert anti-inflammatory effects (18, 19). Abundant studies have shown that IL-27 and IL-35 produced by multiple immune cells were essential for host defense against MTB infection. For example, the expression level of IL-27 in the sputum and plasma of patients with PTB was higher when compared to controls (20), and the serum IL-35 level in patients with PTB was also increased (14). To further assess the roles of *IL27* and *IL-35* genes in the pathogenesis of PTB, we focused on the analysis of the influence of *IL27* and *IL-35* gene polymorphisms on the susceptibility to PTB and several major clinical manifestations.

In the process of MTB infection, IL-27 cooperating with IL-12 activated Th1 cells against MTB infection through the JAK/STAT1 pathway, and IL-27 could also induce the activation of Tr1 cells by the JAK/STAT3 pathway, thereby inhibiting the host immune response (12). In addition to serum, the IL-27 concentration in sputum was confirmed to be positively associated with mycobacterial load (20). In addition, IL-27 was considered to be a potential biomarker for the diagnosis of PTB and TB pleurisy by comparing its expression level in pleural effusion and bronchoalveolar lavage fluid (21, 22). Notably, the polymorphism in the *IL-27* gene could regulate host innate and adaptive immune response and was associated with infectious diseases, autoimmune diseases, and caner (23–25). For example, the rs17855750 variant was considered to be a risk factor for papillary thyroid cancer in the Chinese population (23), and a protective factor for ulcerative colitis in the Mexican population (24), while rs153109 might contribute to virus clearance in hepatitis C virus (HCV) infection (25). However, our present study revealed no association between rs153109 and rs17855750 polymorphism in the *IL-27* gene and susceptibility to PTB. This was consistent with the study by Oh et al., and they suggested that the rs17855750, rs181206, rs153109, and rs181207 variants within the *IL-27* gene were unrelated to PTB risk in a Korean population (26). In contrast, rs17855750 polymorphism was found to be significantly related to PTB risk (18). The differences in these studies might be due to factors such as different sample sizes, ethnic differences, host–pathogen interactions, and gene–environment interactions. However, these differences also reminded us that *IL-27* gene polymorphism might be involved in PTB susceptibility, while more replication studies were still needed.

As an important anti-inflammatory cytokine, IL-35 was produced by B cells, regulatory T cells, monocytes, and dendritic cells, and played key roles in many immune-related diseases (27). The involvement of IL-35 in MTB infection had attracted more attention; for example, serum IL-35 level was significantly increased in patients with TB pleural effusion when compared to those with malignant pleural effusion (28). The study by Jiang et al. found that the expression level of IL-35 in peripheral blood mononuclear cells and Treg cells of patients with PTB was significantly increased compared to that in normal controls, and the increased IL-35 level in Treg cells could enhance their inhibitory effect among MTB infection (29). Further analysis showed that IL-35 inhibition resulted in a decrease in the number of Treg cells and an increase in the number of Th1 cells (29). This implied that IL-35 was closely involved in the pathogenesis of PTB; hence, it was very necessary to explore the relationship between *IL-3*5 gene variation and PTB genetic susceptibility. Previous studies found that the *IL-3*5 rs4740 variant showed a significant relationship with a decreased risk for PTB (30), while the rs568408 variant was not related to PTB risk (31). Nevertheless, our study found no significant association between rs2243123, rs2243135, rs568408, rs428253, rs4740, and rs9807813 variants in the *IL-35* gene and PTB susceptibility. Interestingly, our study found that the GAC haplotype (rs428253–rs4740–rs9807813) in the *IL-35* gene was associated with PTB susceptibility and was a protective factor affecting PTB development, which indicated that *IL-35* gene variation had an important role in PTB susceptibility. This study also analyzed whether the *IL-35* gene variant affected PTB susceptibility by altering IL-35 expression level. The results showed that there was no significant difference in IL-35 plasma level between patients with PTB and controls, and the above six SNPs had no significant effect on IL-35 plasma level in patients with PTB. Combined with the above results, we speculated that *IL-35* gene variation might influence PTB susceptibility, but not through a single SNP, which still needed to be verified by further studies.

Some clinical characteristics of patients with PTB would have an important impact on the treatment process and prognosis of this disease, and these clinical manifestations were also affected by certain gene variations. Zhang et al. found that rs7041 and rs3733359 variants in the *GC* gene were statistically associated with pulmonary infection and fever in patients with PTB (32). Our previous study demonstrated that the *IL-13RA1* rs2495636 polymorphism was related to pulmonary infection and drug resistance, and rs3795175 and rs638376 variants in *IL-13RA2* were associated with drug resistance among patients with PTB (10). Analogously, this study revealed a significant association between the *IL-27* rs17855750 GG genotype and fever. Moreover, *IL-35* rs568408 and rs428253 variants were also reported as key factors in the occurrence of DILI and hypoproteinemia in patients with PTB. The above results indicated that *IL-27* and *IL-35* gene variations were significantly associated with some clinical manifestations of patients with PTB and might play a role in the development of this disease, which had positive effects on the selection of appropriate treatment regimens for patients with PTB.

Several limitations of this research should be noted. First, this study was a case–control study, and there were some biases in revealing causality. Second, this study did not exclude the potential influence of environmental factors, and the gene–environment interaction was also not established. Third, the sample size of this study might not be considered adequate, which could influence the power of our results.

In summary, our results firstly demonstrated that the GAC haplotype for rs428253, rs4740, and rs9807813 in the *IL-35* gene was a protective factor for PTB susceptibility in a Chinese Han population, while *IL-27* gene polymorphism was not involved in the susceptibility to PTB. Moreover, *IL-27* and *IL-35* gene variations might contribute to several clinical manifestations of patients with PTB, such as fever, DILI, and hypoproteinemia.

However, further studies should be conducted to assess the precise function of *IL-27* and *IL-35* gene variation in MTB infection.

## Data availability statement

The original contributions presented in the study are included in the article/supplementary material. Further inquiries can be directed to the corresponding authors.

## Ethics statement

This study was approved by the Ethical Committee of Anhui Chest Hospital. The studies were conducted in accordance with the local legislation and institutional requirements. The participants provided their written informed consent to participate in this study.

## Author contributions

LG: Investigation, Methodology, Writing – original draft. YX: Investigation, Methodology, Writing – original draft. YL: Investigation, Writing – review & editing. PH: Investigation, Writing – review & editing. SL: Formal analysis, Writing – review & editing. YX: Investigation, Writing – review & editing. H-MW: Methodology, Project administration, Writing – review & editing. QH: Data curation, Formal analysis, Methodology, Writing – review & editing. HW: Methodology, Project administration, Writing – original draft.
